# Seasonal dynamics of soil CO_2_ efflux across land use systems and implications for mitigation under a changing climate

**DOI:** 10.1038/s41598-026-50708-7

**Published:** 2026-07-28

**Authors:** Famoussa Dembélé, Stephen Adu-Bredu, Reginald Tang Guuroh, Eunice Okyere Agyapong, Padmore Boateng Ansah, Nat Owusu-Prempeh, Bismark Owusu, Dzigbordi Solomon-Ayeh, Suleiman Usman Yunusa, Aboubakar Bengaly, Larissa Raatz, Roman Hinz, Atinuke Adebanji, Samuel Kingsley Oppong, Rüdiger Schaldach, Amanuel Woldeselassie Gebremichael, Anja Linstädter

**Affiliations:** 1 WASCAL Graduate Research Program on Climate Change and Land Use, Kwame Nkrumah University of Science and Technology, Kumasi, Ghana; 2 Département des Sciences et Techniques Agricoles, Institut Polytechnique Rural de Formation et de Recherche Appliquée, Koulikoro, Mali; 3 CSIR-Forestry Research Institute of Ghana, KNUST, P.O. Box UP 63, Kumasi, Ghana; 4 Department of Forest Resources, Kwame Nkrumah University of Science and Technology, PMB KNUST, Kumasi, Ghana; 5 Department of Natural Resources Management, CSIR – College of Science and Technology, Box UP 63, Kumasi, Ghana; 6 Department of Renewable Natural Resources, Kwame Nkrumah University of Science and Technology (KNUST), PMB KNUST, Kumasi, Ghana; 7 Department of Agricultural and Bioresources Engineering, Ahmadu Bello University, Zaria, Nigeria; 8 Science Management Unit, Leibniz-Institut für Agrartechnik und Bioökonomie e.V, 14469 Potsdam, Germany; 9 Kassel Institute for Sustainability, University of Kassel, Kassel, Germany; 10 Department of Statistics and Actuarial Science, Kwame Nkrumah University of Science and Technology (KNUST), PMB KNUST, Kumasi, Ghana; 11 Department of Wildlife & Range Management, Faculty of Renewable Natural Resources, Kwame Nkrumah University of Science and Technology (KNUST), PMB KNUST, Kumasi, Ghana; 12 GFZ German Research Center for Geosciences, 14473 Potsdam, Germany; 13 Biodiversity Research/Systematic Botany, University of Potsdam, 14469 Potsdam, Germany; 14 Département de l’Information Environnementale, Agence de l’Environnement et du Développement Durable (AEDD), Bamako, Mali

**Keywords:** Soil respiration rate, Land-use type, Climate change mitigation, Tropical Forest ecosystems, Semi-deciduous forest, Carbon cycle, Agroecology

## Abstract

Carbon dioxide (CO_2_) is a major greenhouse gas driving climate change. In Ghana, the Agriculture, Forestry, and Other Land Use (AFOLU) sector remains a significant source of CO_2_ emissions, largely due to land use change and degradation. This study assessed seasonal dynamics of soil respiration rates (SRR) across four land-use types, forest, fallow, maize, and rice fields, within the semi-deciduous forest zone of Ghana. The aim was to provide baseline data and identify key soil and environmental factors influencing SRR across these systems. SRR was measured twice monthly over 13 months using a closed-chamber system, with concurrent measurements of soil moisture and temperature, while baseline soil properties (organic matter, pH, and texture) were determined from initial soil sampling. Correlation and stepwise regression analyses were performed to determine the variables most strongly associated with SRR. Results revealed clear temporal and land-use differences, although seasonal patterns were not uniform across sites. Fallow land and croplands (maize and rice fields) recorded the highest SRR values within the study area, whereas forest plots consistently showed the lowest efflux, largely due to persistent moisture limitation rather than temperature or substrate availability. Soil OM, pH, moisture, and silt content were the most influential predictors of SRR, with the final regression model explaining 58% of the observed variability. These findings highlight the importance of forest conservation and sustainable land management in mitigating CO_2_ emissions in tropical regions. To further reduce emissions in the AFOLU sector, policies should also support reforestation, agroforestry, and reduced soil disturbance, which enhance soil carbon storage and promote sustainable land use.

## Introduction

Climate change is one of the most pressing global environmental challenges of the 21 st century, driven primarily by the increasing concentration of greenhouse gases (GHGs) in the atmosphere, notably Carbon dioxide (CO_2_), methane (CH_4_), and nitrous oxide (N_2_O). Among these, CO_2_ is the most significant, accounting for approximately 76% of global GHG emissions^1^. Anthropogenic activities, including fossil fuel combustion, deforestation and industrial processes, have elevated atmospheric CO_2_ levels, contributing to global warming and altering climatic patterns^2^. Understanding the dynamics of CO_2_ emissions from various sources and sinks is therefore crucial for developing effective mitigation strategies to achieve international climate targets, such as those outlined in the Paris Agreement.

In Africa, CO_2_ emissions are steadily rising due to rapid economic development, urbanisation, and changing land-use practices, though they remain lower than the global average^3^. In West Africa, particularly Ghana, significant increases in CO_2_ emissions have been observed, mainly from the energy sector as well as the agriculture, forestry and other land-use (AFOLU) sector^4^. According to Ghana’s Environmental Protection Agency^4^ report, the country’s national GHG emissions in 2024 were estimated at 51.78 million tonnes of CO_2_ equivalent (51.78 MtCO_2_e), with the energy sector contributing about 51.2%, the AFOLU sector 38.3%, the waste sector 7.3% and the industrial processes and product use (IPPU) sector 3.2%. The AFOLU sector, as the second largest source of emissions, is primarily driven by deforestation, agricultural expansion, and land degradation. The Food and Agriculture Organisation of the United Nations (FAO) has identified agricultural expansion as a significant driver of deforestation and forest degradation, leading to exacerbating GHG emissions^5^.

The AFOLU sector can act as both a source and a sink of CO_2_; however, practices such as slash-and-burn agriculture, forest-to-cropland conversion, and poor land management release substantial emissions. Although the sector has the potential to provide removals under sustainable management, current trends in deforestation and land-use change make AFOLU a net source of CO_2_ in many tropical landscapes^6,7^. Soil respiration (SRR), the process by which CO_2_ is released from the soil surface due to microbial decomposition of organic matter and root respiration, is a significant component of terrestrial CO_2_ fluxes^8^. Globally, SRR contributes an estimated 60–90 Pg C yr^− 1^ to atmospheric CO_2_, approximately ten times greater than emissions from fossil fuels^9^. Understanding SRR processes, particularly in relation to various land-use land-cover (LULC) types, is therefore crucial for determining accurate carbon budgeting and implementing effective climate mitigation in the AFOLU sector.

Monitoring soil CO_2_ emissions is not only important for scientific understanding but also for policy formulation and implementation. Ghana, as a signatory to the Paris Agreement, has committed to reducing GHG emissions through its Nationally Determined Contributions (NDCs)^10^. This includes improving the management of forest and agricultural lands to enhance carbon sequestration. Reliable data on soil CO_2_ fluxes are crucial for refining national emission inventories and monitoring progress toward these international commitments, aligning with global climate goals set forth by the United Nations Framework Convention on Climate Change (UNFCCC).

In Ghana, soil respiration has traditionally been measured using the static alkali absorption method due to its simplicity and low cost^11^. In this approach, CO_2_ emitted from the soil is absorbed by a sodium hydroxide (NaOH) solution and later quantified by titration^12^. Although effective, this method is labour-intensive and prone to human error. Automated systems using infrared gas analysers offer higher temporal resolution but remain uncommon due to cost and logistical constraints^15^. Most studies in Ghana have therefore relied on short-term measurements ranging from several weeks^14,15^ to nine months^13^, which limits the understanding of seasonal SRR dynamics. Long-term monitoring is needed to characterise temporal variability across Ghana’s diverse land-use systems.

Similar challenges in temporal and land-use–specific monitoring of soil respiration have been documented across other tropical and subtropical regions. For example, studies from India’s seasonal wet tropical forests show that soil respiration responds strongly to rainfall and moisture, while its relationship with temperature varies across timescales, reflecting complex environmental controls^16^. Likewise, research in Southeast Asia has reported diverse respiration responses across successional forest stages, highlighting the need for site-specific and long-term measurements^17^. These findings are similar to the situation in Ghana, where long-term, land-use specific SRR data remain scarce. Together, they underscore the importance of comprehensive measurements across tropical biomes to better capture spatial and temporal variability in soil respiration.

Soil respiration is a complex biochemical process governed by various environmental and physico-chemical factors, with climatic conditions playing a critical role. Among these, temperature and moisture are key regulators of SRR as they directly influence microbial activity and root respiration. Elevated temperatures enhance microbial metabolic rates and enzymatic activity, resulting in accelerated organic matter decomposition and subsequent CO_2_ release^18^. However, this effect is moderated by soil moisture; both excessively dry and waterlogged conditions can inhibit microbial activity and reduce soil respiration^19^. In addition to climatic conditions, soil physico-chemical properties such as pH, texture, and soil organic matter (SOM) content are crucial in determining soil respiration. Higher SOM provides more substrate for microbial decomposition, thereby increasing SRR as microbes break down the organic material into simpler compounds, releasing CO_2_ in the process^8^. Similarly, soil pH affects the composition and activity of microbial communities, while soil texture influences aeration and moisture retention, both of which are critical for microbial and root respiration processes^20^. Understanding the interplay between these variables is essential for accurately predicting SRR across different LULC types in Ghana’s semi-deciduous forest zone.

This study, therefore, aims to provide baseline data on SRR from forest, fallow (open forest), and cropland (maize and rice) in Ghana’s semi-deciduous forest zone. Specifically, the study addresses the following research questions: (1) How does SRR respond to temporal changes in weather conditions? (2) How does LULC, topography, and habitat types influence SRR? And (3) Across different land uses, what are the key soil physical and environmental attributes that influence/drive SRR? To answer these questions, a combination of field measurements and laboratory analyses was employed.

## Materials and methods

### Site description and plot selection

**Fig. 1:**
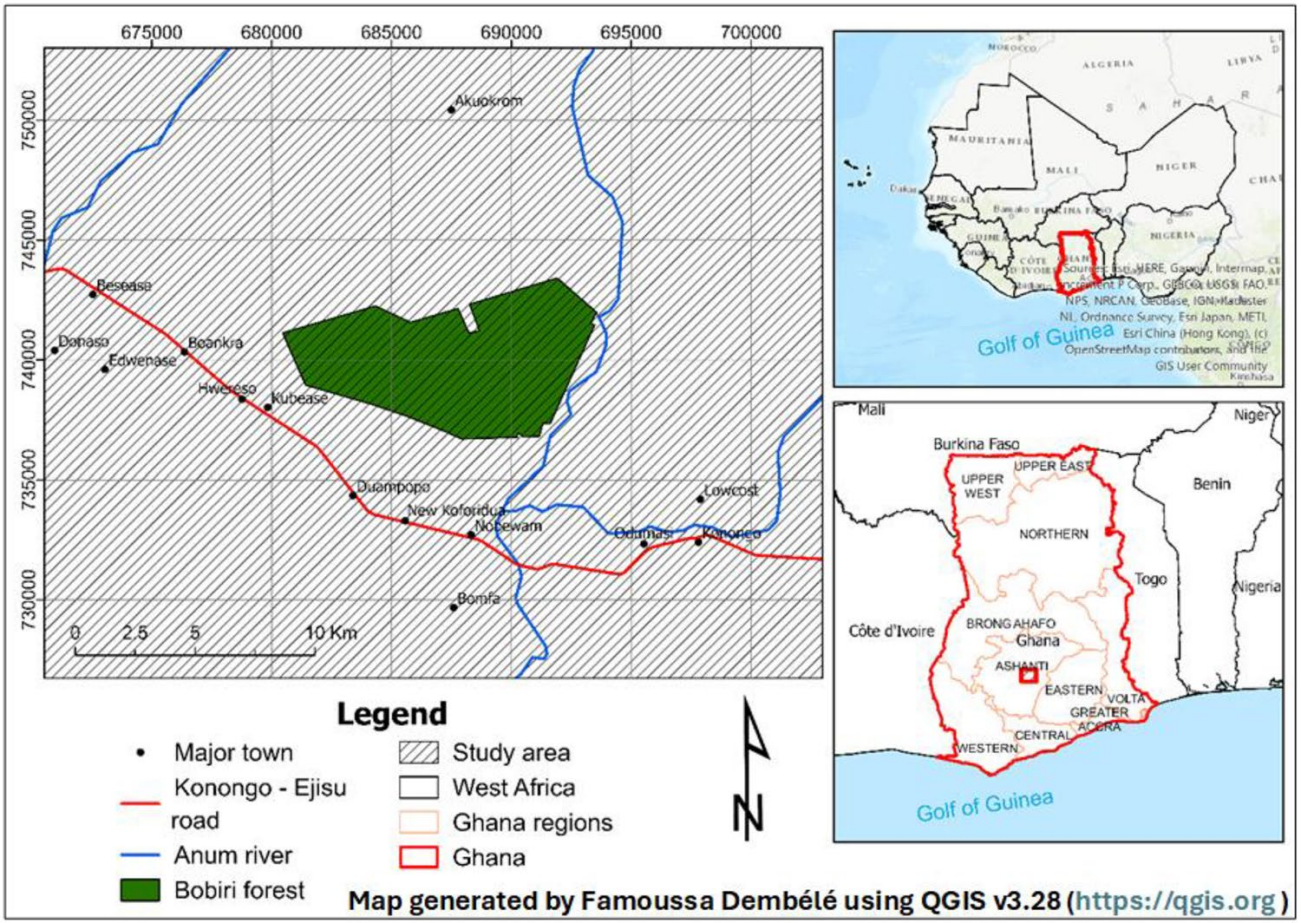
Map of the study area^22^.

The research was conducted within the Bobiri Forest Reserve (BFR) and its surrounding areas in the moist semi-deciduous forest of Ghana (Fig. 1). The study area spans approximately 853 km^2^, and includes the BFR (54 km^2^) and its surrounding communities within the Ejisu and Juabeng Municipalities in the Ashanti region. The BFR is divided into management units designated for production, tourism, research and conservation purposes, featuring notably landmarks such as butterfly sanctuary and a strict nature reserve^21^.

The region experiences a humid semi-equatorial climate with a bimodal rainfall pattern. The major rainy season spans April to July, followed by a minor rainy season from September to November. A long dry season extends from December to March, with a brief dry spell in August. Climatic data collected from the Ghana Forestry Research Institute (CSIR-FORIG), approximately 35 km from the BFR, indicates annual rainfall ranging from 1,189 mm (2023) to 1,450 mm (2018), with a mean of 1,441.64 ± 215.43 mm yr^− 1^. The mean annual temperature during this period was 24.65 ± 0.67 °C, with August being the coolest month (22.48 ± 3.62 °C) and February being the hottest (25.66 ± 3.10 °C).

Four major land-use types, closed forest, open forest, cropland and mixed vegetation, were identified from the 1986, 2007, and 2022 satellite classifications^22^. For the present field study, representative plots were selected as follows:

- closed forest: forest plots located within the BFR’s (dense canopy, minimal disturbance)^21^;
- open forest: fallow plots comprising secondary vegetation dominated by shrubs, grasses, and scattered trees that naturally regenerated after previous cultivation (left uncultivated for 7 to 15 years).
- cropland: actively cultivated maize and rice fields managed under different fertilisation regimes^23^.

This classification captures the distinct agricultural practices and land management strategies that influence soil processes and ecosystem dynamics in the region.

#### Definition of seasons in this paper

The study area lies within Ghana’s moist semi-deciduous forest zone, which experiences a bimodal rainfall pattern typical of the humid semi-equatorial climate. Based on long-term meteorological records (FORIG station, 2018–2023), rainfall and temperature patterns delineate two main seasons:

- wet season: April to October, comprising the major rainy season (April – July) and the minor rainy season (September – October).
- dry season: November to March, with a brief harmattan period (December – February) characterised by low rainfall, high temperatures, and reduced humidity.

Accordingly, monthly field measurements were grouped into wet and dry seasons for comparative analysis of soil respiration, soil moisture, and temperature dynamics.

### Land-use selection and experimental layout

**Fig. 2:**
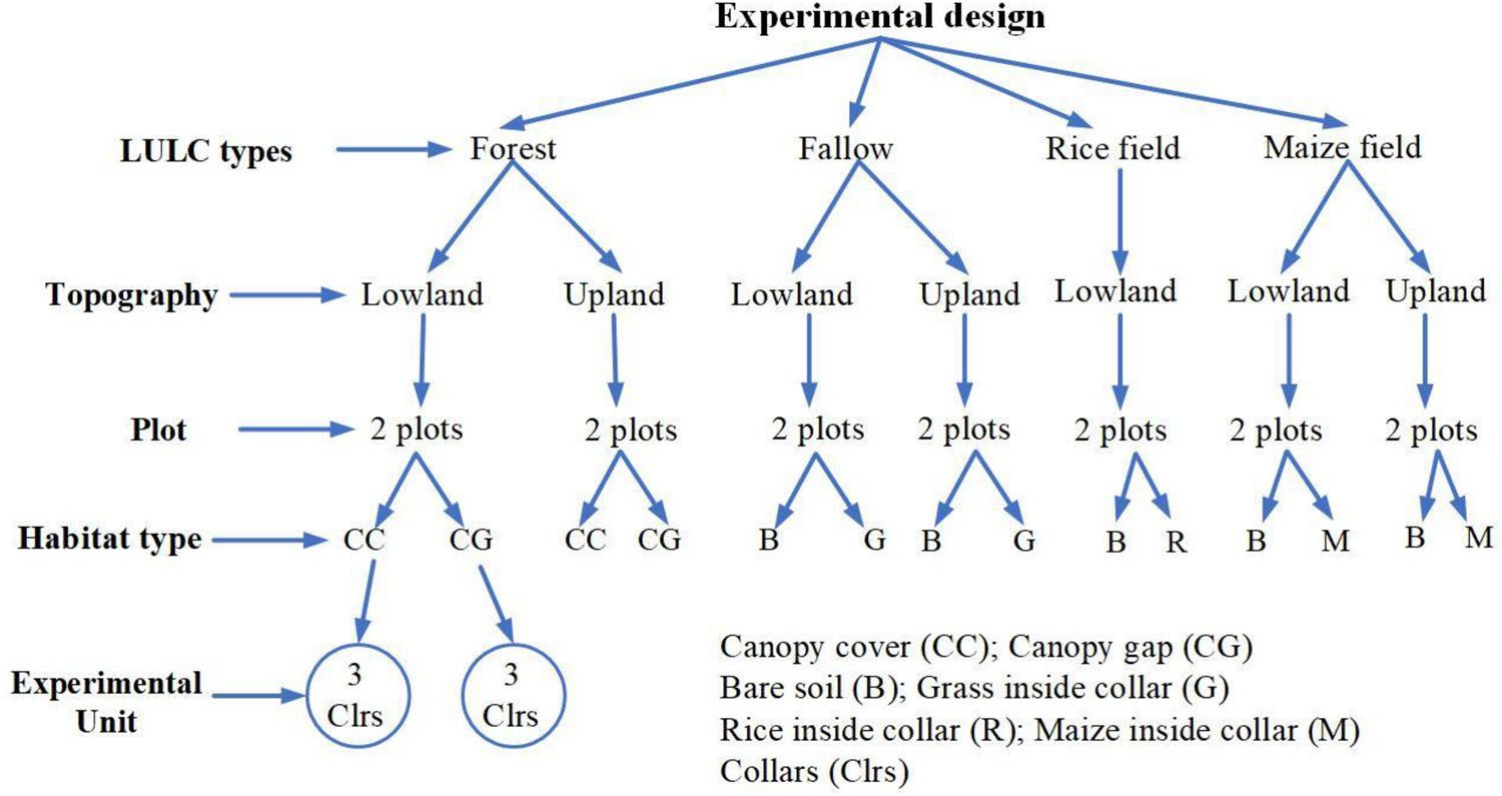
Experimental design for measuring soil respiration.

A total of 14 plots, each measuring 50 × 50 m, were established across four land-use types: forest, fallow, maize, and rice fields to assess SRR. The experimental design incorporated topographical variation and habitat types. For each land-use type, two plots representing upland (UP) and lowland (LL) areas were selected, except for rice fields, as rice cultivation primarily occurs in lowlands. To ensure sufficient heterogeneity within each land-use types, plots were spaced at least 200 m apart. Within each plot, two dominant habitat types were identified, and three PVC collars (25 cm in diameter, 15 cm in height) were installed per habitat type for SRR measurements. The selected habitat types varied by land-use: forest plots included bare soil canopy cover (shaded) and bare soil in canopy gap (exposed); fallow plots included bare soil and grass-covered areas; and maize and rice fields included bare soil and areas with crops. This comprehensive experimental layout design enabled a detailed assessment of SRR across diverse land-use types, topographical gradients, and habitat types, ensuring robust data collection and analysis (see Fig. 2 for the experimental design).

### Data collection, preprocessing and analyses

#### Data collection

##### Soil carbon dioxide measurement

Soil respiration rate (SRR) was measured twice a month at two-week intervals over 13 months (June 2023 – June 2024) across all selected land-use types. A closed chamber method was employed to measure SRR across the selected land-use types. To minimize soil disturbances, PVC collars were installed 72 h prior to the start of measurements and remained in the field for the entire study period. These collars were inserted into the soil, leaving approximately 5 cm above the surface to allow proper chamber attachment and minimize disturbance of the surrounding soil. Each plot had six collars installed, with three collars allocated per habitat type.

**Fig. 3:**
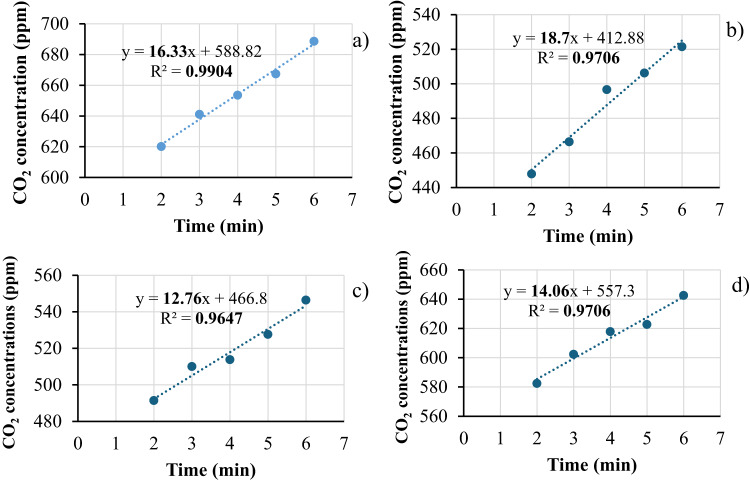
Raw CO_2_ concentration measured at 1-minute intervals for (**a**) Forest, (**b**) Fallow, (**c**) Maize, and (**d**) Rice plots. The slope shown is the initial rate in ppm min^− 1^, which is later converted to ppm h^− 1^ for flux calculations.

Respiration chambers, made of PVC with diameters matching the collars, were used in two heights (40 cm and 100 cm) depending on the habitat type. Each chamber was equipped with one or two fans to homogenize air inside. Sensors mounted on top of the chambers measured SRR, air temperature, air pressure, and relative humidity. A CO_2_ sensor (Vaisala GMP252, range 0–10,000 ppm) recorded CO_2_ concentrations, while the Tekbox TBSHTP05 sensor measured relative humidity (± 2%, 0–100%), air temperature (–20 °C to + 55 °C), and barometric pressure (± 0.5 hPa, 300–1250 hPa). All sensors were connected to a GP2 datalogger, which recorded data every minute for seven minutes following chamber closure. To ensure accuracy, data from 2nd to 6th minutes (five data points) were analysed, discarding the first and last minutes to avoid noise. Linear regression was applied to calculate the slope, and R^2^ values of ≥ 90% were only used to ensure data quality (Fig. 3^24^;). Simultaneously, volumetric soil moisture and temperature were measured using a WET 150 soil probe inserted at a depth of 5 cm near each collar.

##### Soil sampling and physico-chemical analyses

Soil samples were collected from all 14 plots to analyse physico-chemical properties, following a habitat-based sampling design. Two sampling methods, composite and single, were collected from the top 10 cm of soil to ensure consistency across plots. For composite samples, three cores were taken near the collars within each habitat type, these were thoroughly mixed to form a uniform sample, and 1.0 kg subsample was taken to the laboratory for physico-chemical analyses. Single samples, on the other hand, comprise individual soil samples collected using a soil core from each habitat type, also adjacent to the collars for bulk density measurement. Fresh weights were measured and recorded immediately after collection to maintain accuracy and ensure reliable subsequent analyses.

##### Physico-chemical analysis

The physical properties of soil, including soil water content, bulk density (BD), total soil porosity (TSP), and water-filled pore space (WFPS), were measured once during the study period and analysed using standard protocols. Soil water content was determined by drying samples at 105 °C until a constant weight was achieved, following the method outlined by^24^. Bulk density was calculated by measuring the mass of oven-dried soil per unit volume, which helped in estimating total soil porosity using the relationship between bulk and particle density. Water-filled pore space was derived from the soil moisture content recorded from the soil moisture probe 150 WET sensor and total porosity, indicating the degree of soil aeration and water retention capacity^25^. Soil texture was determined using the hydrometer method, which involved dispersing soil particles in a sodium hexametaphosphate solution and measuring sedimentation rates^26^.

The chemical properties of soil, such as pH, total nitrogen (TN), organic carbon (C), organic matter, available phosphorus (P-avail), and exchangeable cations (Ca, Mg, K, Na), were also measured once to determine the baseline situation and assessed using well-established methods. Soil pH was measured in a 1:2.5 soil-water suspension using a pH meter^15^. Total nitrogen was determined via the Kjeldahl method, which involves acid digestion, distillation, and titration^27^. Organic carbon was analysed using the Walkley-Black wet oxidation method, and organic matter content was derived by multiplying organic carbon by 1.724 (Van Bemmelen factor)^28^. Available phosphorus was extracted using the Bray-1 method, where phosphate was reacted with ammonium molybdate and ascorbic acid and measured spectrophotometrically^29^. Exchangeable cations were extracted with 1 N ammonium acetate (NH_4_OAc) and quantified using flame photometry (K, Na) and EDTA titration (Ca, Mg)^30^. These analyses provide essential insights into soil fertility and nutrient availability.

#### Data preprocessing

##### Soil respiration rate calculation

The soil respiration rate (SRR), expressed in kilograms per hectare per day, was calculated using the static chamber system and the following equation developed by^31,32^

Where:

- $\:F$= Flux in kg CO_2_ ha^− 1^ d^− 1^.
- $\:{\alpha\:}_{v}$ = Volume-based CO_2_ slope (ppm h^− 1^).
- $\:M$= Molecular weight of CO_2_ = 44.01 g mol^− 1^.
- $\:P$= Atmospheric pressure = 1,010 × 103 Pa.
- $\:R$= Universal gas constant = 8.314 J mol^− 1^ K^− 1^.
- $\:T$= Field temperature in Kelvin (℃ + 273).
- $\:V$= Total headspace volume (m^3^).
- $\:A$= Soil surface area covered by the collar (m^2^).
- $\:0.0024$= The conversion factor from hrs to days and m^2^ to ha.

The flux ($\:{\alpha\:}_{v})$ is obtained by calculating the initial slope of CO_2_ concentration over time. This raw slope, recorded in ppm min^− 1^, is then converted to ppm h^− 1^ before being used in the flux equation. The final flux computation incorporates atmospheric pressure, temperature, the molecular weight of CO_2_, the chamber volume, and the soil surface area. The conversion factors in Eq. (1) account for the transformation of time (hours to days) and area (m^2^ to ha). Figure 3 presents the raw CO_2_ data used during initial screening, including the exclusion of slopes with R^2^ values below 80%, prior to slope conversion and flux calculation.

##### Soil respiration model development

Soil respiration was modelled using both linear and nonlinear regression approaches. A stepwise multiple regression analysis was conducted with four predictor variables, encompassing soil biophysical properties and environmental factors. Three model types linear, exponential and logarithmic were employed. The predictor variables were selected based on their strong relationships with soil respiration and the detailed model structures are presented in Table 1. To evaluate the performance of these models in estimating soil respiration rates, three statistical indicators were used: the Akaike Information Criterion (AIC), the coefficient of determination (R^2^), and percentage bias (%Bias).

The AIC assesses the trade-off between model accuracy and complexity by penalizing overfitting, with lower AIC values indicating a better-fitting model^33^. The R^2^ quantifies the proportion of variance in the observed data explained by the model, with values closer to 1 reflecting greater explanatory power^34^.

Percentage bias measures the systematic deviation between predicted and observed values, where values near 0 indicate minimal bias, positive values suggest overestimation and negative values indicate underestimation^35^. These statistical indicators collectively provide a comprehensive evaluation for model performance, ensuring accuracy, efficiency, and reliability in selecting the optimal model for estimating soil respiration.

**Table 1:** Equations of multiple linear and non-linear regression.

Model types	Equation	References	Eq
Linear	$\:y={\beta\:}_{0}+{\beta\:}_{1}{x}_{1}+\:{\beta\:}_{2}{x}_{2}+\dots\:{\beta\:}_{n}{x}_{n}+\epsilon\:$ Where $\:{\beta\:}_{0},\:{\beta\:}_{1},\:{\beta\:}_{2}$ and $\:{\beta\:}_{n}$​ are coefficients for the predictor $\:{x}_{i}$, and $\:\epsilon\:$ is the error.	^(36)^	2
Exponential	$\:y=a{e}^{({\beta\:}_{1}{x}_{1}+\:{\beta\:}_{2}{x}_{2}+\:\cdots\:+{\beta\:}_{n}{x}_{n})}$ Where *a* is a constant, $\:{\beta\:}_{0},\:{\beta\:}_{1},\:{\beta\:}_{2}$ and $\:{\beta\:}_{n}$​ are coefficients for each predictor $\:{x}_{i}.$	^(37)^	3
Logarithmic	$\:y={\beta\:}_{0}+\:{\beta\:}_{1}\mathrm{log}\left({x}_{1}\right)+\:{\beta\:}_{2}\mathrm{log}\left({x}_{2}\right)+\dots\:{\beta\:}_{n}\mathrm{log}\left({x}_{n}\right)$ Where $\:{\beta\:}_{0},\:{\beta\:}_{1},\:{\beta\:}_{2}$ and $\:{\beta\:}_{n}$​ are coefficients for the predictors.	^(38)^	4

#### Data analyses

The data was log-transformed to perform a factorial analysis of variance (ANOVA), which was used to determine the effect of seasonal variation on SRR across the four land-use types. Additionally, a two-sample t-test (assuming unequal variance) was conducted to determine significant differences in SRR, soil moisture and temperature between the wet and dry seasons across the different land-use types.

A multifactorial ANOVA was also conducted to assess the influence of land-use types, topography and habitat types on SRR. Tukey’s honestly significant difference (HSD) test was then employed to identify significant differences among the means at a 5% significance level. To explore the relationships between physico-chemical parameters and SRR, a pairwise correlation matrix was computed. This informed the selection of predictors for subsequent multiple linear and nonlinear regression analyses. Variables such as soil moisture, temperature, pH, organic matter and texture were evaluated for their effects on SRR. All statistical analyses were performed using R statistical software (version 4.4.1)^39^, Excel and SAS OnDemand for Academics.

## Results

### Seasonal variation in soil respiration rate, moisture and temperature

**Fig. 4:**
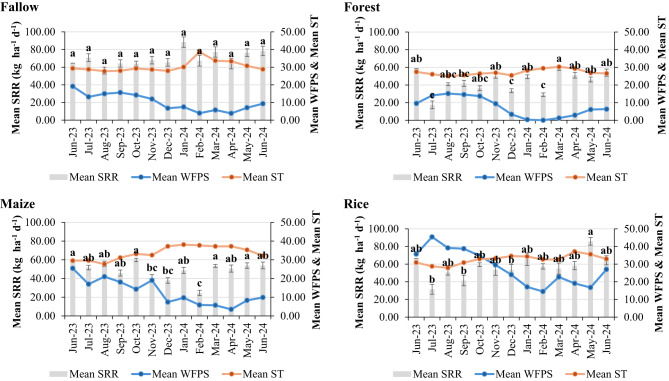
Seasonal variation in soil respiration rate (SRR), temperature, and WFPS across Fallow, Forest, Maize, and Rice lands. Different letters indicate significant differences (*p* < 0.05); error bars show standard errors.

Seasonal variation in this study refers to the difference between the wet season (April – October) and the dry season (November – March), following the rainfall pattern typical of Ghana’s moist semi-deciduous forest zone. Figure 4 shows monthly changes in SRR, soil temperature (ST), and soil moisture expressed as water-filled pore space (WFPS) for the four land-use types.

Overall, SRR showed irregular and weak seasonal changes rather than a clear wet-dry pattern. In most land uses, SRR peaks occurred at different times, for example, during January – March in fallow and forest plots (88.36 and 58.82 kg CO_2_ ha^− 1^ d^− 1^, respectively), and during May – June in rice and maize fields (85.80 and 57.17 kg CO_2_ ha⁻¹ d⁻¹, respectively). This suggests that short-term changes in soil temperature and moisture had a stronger effect on CO_2_ release than the general seasonal cycle. Soil temperature remained relatively stable throughout the year, while soil moisture followed rainfall patterns, increasing during the wet months and declining in the dry period.

Although rainfall in the area follows a clear bimodal pattern, soil CO_2_ release did not respond in a simple seasonal way. The weak pattern may be due to local conditions such as vegetation cover, land management, and fast wetting-drying cycles of the soil. After rainfall, the soil often dries quickly, creating short bursts of respiration rather than a long seasonal trend. In addition, temperature differences between seasons are small in this tropical climate, so moisture availability becomes the main factor controlling respiration. These results show that soil CO_2_ efflux in the semi-deciduous forest zone is mainly driven by short-term soil conditions rather than by broad seasonal changes.

### Effect of topography on soil respiration rate

**Fig. 5:**
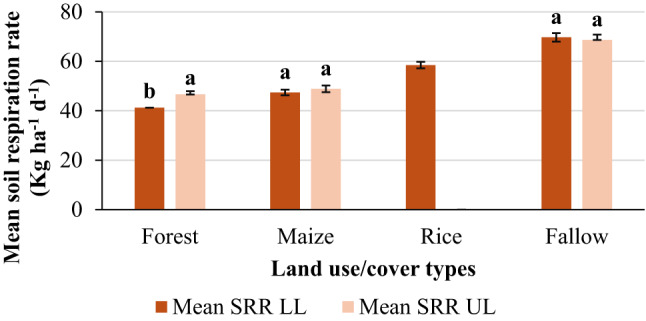
Effect of topography (upland vs. lowland) on soil respiration rate across land-use types.

The effect of topography on soil respiration rate (SRR) was assessed within each land-use type (Fig. 5). The results show the mean SRR ± standard error and corresponding significance letters for upland and lowland plots.

In forest plots, the mean SRR was significantly higher in the upland (46.61 ± 1.31 kg CO_2_ ha^− 1^ d^− 1^; letter a) than in the lowland (41.25 ± 1.16 kg CO_2_ ha^− 1^ d^− 1^; letter b), indicating a clear topographic effect. The slightly larger standard error in the upland suggests greater spatial variability in respiration.

In fallow plots, SRR did not differ significantly between upland (68.68 ± 2.12) and lowland (69.73 ± 1.79) areas, both marked with the same significance letter (a). The smaller standard error in the lowland indicates more homogeneous fluxes.

Similarly, maize fields showed no significant topographic difference, with mean SRR of 48.85 ± 1.35 (upland (UL)) and 47.40 ± 1.31 (lowland (LL)). For rice fields, measurements were available only in lowland sites, yielding a mean SRR of 58.48 ± 1.72.

Overall, lowland plots showed lower variability in SRR, as indicated by smaller standard errors, whereas upland sites exhibited greater heterogeneity, particularly in fallow and forest land-use types. The effect of topography on SRR was not statistically significant (F = 2.999, *p* = 0.083), suggesting that topography plays only a minor role in controlling soil respiration under the environmental conditions of the study area.

### Extent of change from the previous month

Soil respiration rates (SRR) varied across land use types from July 2023 to June 2024. Excluding fallow land, SRR declined from June to July 2023 in forest, maize, and rice lands, with the most significant decrease in the forest (−70.17%). This trend reversed in August, showing sharp increases, particularly in forest (134.29%) and rice (60.58%). Fallow land initially increased (9.58%) in July before declining (−20.70%) in August. Between August and September 2023, forest and fallow SRR increased, while maize and rice decreased. However, in September – October 2023, forest and fallow declined, whereas maize and rice rose. A similar alternating pattern continued in October – November 2023, with forest and fallow increasing, while maize and rice declined.

**Fig. 6:**
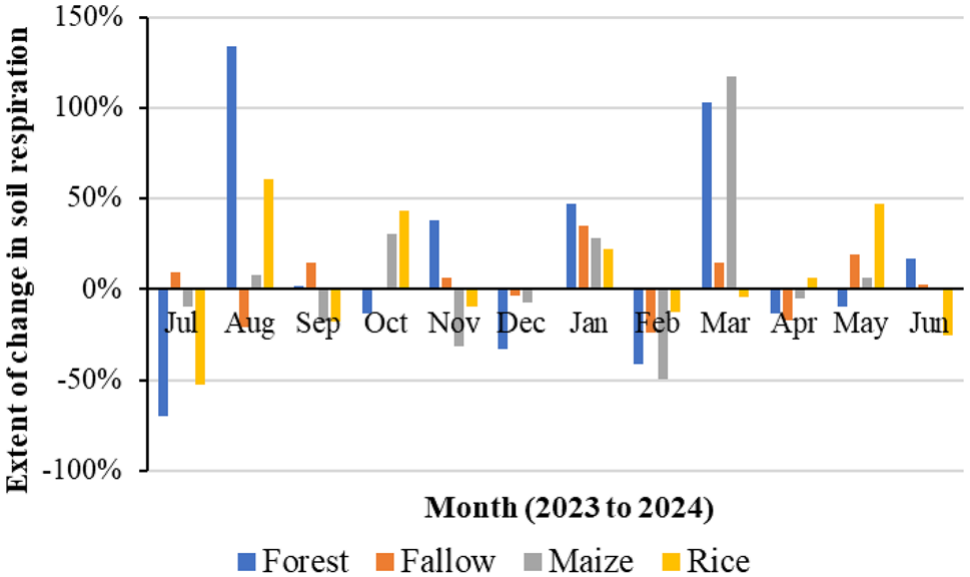
Extent of change in soil respiration rate from that of the previous month in the four land uses.

From November to December 2023, all land-uses showed reductions in SRR, followed by a notable increase from December to January 2024, particularly in forest (46.98%). In February 2024, SRR declined sharply, particularly in maize (– 49.56%) and forest (– 41.14%) plots, followed by a general increase in March 2024, except for rice. This decline coincided with a marked reduction in soil moisture, as WFPS reached its lowest values across all land-use types (below 10% in maize and forest plots). Meanwhile, soil temperature remained high and relatively constant during this period. These conditions suggest that the dominant environmental stressor in February was low soil moisture, which limited microbial and root respiration, while the increase in March reflected a recovery of SRR following rewetting of the soil. From April to May 2024, only forest decreased, while in May – June 2024, only rice exhibited a reduction. Both soil moisture (WFPS) and soil temperature (ST) showed temporal fluctuations; however, ST varied little throughout the year, whereas soil moisture fluctuated markedly. Despite these changes, neither variable consistently aligned with the observed SRR patterns across the different land-use types (Fig. 6).

### Soil characteristics

The variation in soil nutrients and physicochemical properties across different LULC types reveals significant differences in organic matter content, bulk density, pH and total nitrogen. Organic matter was highest in fallow soils (3.48 ± 0.15%) and forest soils (3.19 ± 0.13%), while lower levels were observed in maize fields (1.56 ± 0.07%) and rice fields (2.48 ± 0.15%). Bulk density was lowest in forest (1.25 ± 0.05 g cm^− 3^) and fallow soils (1.24 ± 0.05 g cm^− 3^) but higher in maize (1.39 ± 0.06 g cm^− 3^) and rice fields (1.40 ± 0.09 g cm^− 3^), indicating greater soil compaction in cultivated fields. Soil pH was slightly acidic across all LULC types, with the highest value recorded in rice fields (6.01 ± 0.36), followed by fallow (5.73 ± 0.25), maize (5.66 ± 0.24), and forest soils (5.47 ± 0.22). Similarly, the total nitrogen content was highest in forest soils (0.23 ± 0.01%) and fallow soils (0.19 ± 0.01%), while maize fields (0.11 ± 0.005%) and rice fields (0.10 ± 0.006%) had the lowest values. Overall, forest and fallow lands maintain higher levels of organic matter and nitrogen, whereas agricultural fields exhibit lower values and higher bulk density, likely due to cultivation practices and soil compaction. These results are illustrated in Table 2.

**Table 2:** Soil Chemical and Physical Characteristics Across Land-Use Types (0–10 cm Depth).

Soil parameters	Land-use type				Pr(> F)
	Fallow	Forest	Maize	Rice	
EC	395.21^**a**^	345.4^a^	324.89^a^	141.02^b^	2.27e^− 06*^
pH	5.73^**b**^	5.47^c^	5.66^b^	6.01^a^	2e^− 16 *^
Phosphorus (mg kg^− 1^)	7.62^**c**^	15.04^a^	12.25^b^	7.1^c^	2e^− 16 *^
TN (%)	0.19^**b**^	0.23^a^	0.11^c^	0.1^c^	2e^− 16 *^
Ava. P (mg kg^− 1^)	47.06^**b**^	86.58^a^	0.13^c^	0.1^c^	2e^− 16 *^
Organic matter (%)	3.48^**a**^	3.19^b^	1.56^d^	2.48^c^	2e^− 16 *^
Bulk density (g cm^− 3^)	1.24^b^	1.25^b^	1.39^a^	1.4^a^	2e^− 16 *^
sand (%)	77.53^b^	88.75^a^	77.7^b^	77.65^b^	2e^− 16 *^
clay (%)	8.89^c^	4^d^	11.18^a^	10.36^b^	2e^− 16 *^
silt (%)	13.57^a^	7.26^c^	11.12^b^	11.99^b^	2e^− 16 *^
Texture	Sandy loam	Sand	Sandy loam	Sandy loam	

*Note***: **Electrical conductivity (EC, µS cm^-1^), soil acidity (pH), Total nitrogen (TN, %), Available phosphorus (Ava. P, mg kg^-1^)

### Interaction between respiration rate and land-use

The ANOVA results confirm significant effects of land-use type, month, and their interaction on soil respiration rates (SRR) over the 13 months (June 2023 – June 2024) (Table 3). Land-use type had an influence (F = 119.355, *p* < 0.001), indicating significant variation in SRR across different land-use systems. Monthly variations were also significant (F = 13.507, *p* < 0.001), suggesting that seasonal factors such as soil moisture and temperature play a crucial role in regulating respiration rates. Additionally, the interaction between land-use and month (F = 4.352, *p* < 0.001) shows that the effect of land-use on SRR fluctuates throughout the year, likely due to differences in land management practices, crop growth cycles, and environmental conditions. For example, rice fields may exhibit higher SRR during wet months due to flooding and increased microbial activity, while forest and fallow lands may show peak emissions during periods of high biomass decomposition. In the rice fields, SRR did not increase directly with soil moisture (WFPS) but instead showed an inverse pattern during several months. In June 2023, SRR was moderate (66.40 kg CO_2_ ha^− 1^ d^− 1^) at a WFPS of 35.65%, while in July 2023, SRR declined sharply to 31.46 kg CO_2_ ha^− 1^ d^− 1^ as WFPS increased to 45.38%, indicating that higher soil moisture may have restricted aerobic microbial respiration. The highest SRR (85 kg CO_2_ ha^− 1^ d^− 1^) was recorded in May 2024 when WFPS was relatively low (16.74%), suggesting enhanced CO_2_ release under well-aerated soil conditions. Between May and June 2024, SRR decreased to 64.30 kg CO_2_ ha^− 1^ d^− 1^ as WFPS rose to 26.97%, even though soil temperature remained nearly stable. These observations indicate that optimal SRR in rice fields occurred under moderately dry conditions, while excess moisture reduced CO_2_ efflux, likely by limiting oxygen availability for aerobic microbial activity.

**Table 3:** Analysis of variance for soil respiration rate by land-use type and month.

Source	Df	Sum Sq	Mean Sq	F value	Pr (> F)
LUT	3	209,838	69,946	119.355	< 2e^− 16 *^
Months	12	94,989	7,916	13.507	< 2e^− 16 *^
LULC × Months	36	91,819	2,551	4.352	3.44e^− 16 *^
Residuals	1915	1,122,250	586		

Df: degree of freedom, Sum Sq (Sum of Squares), Mean Sq (Mean Square), sig. 0.05 (*).

### Effect of land-use change, topography and habitat types on soil respiration rate

**Fig. 7:**
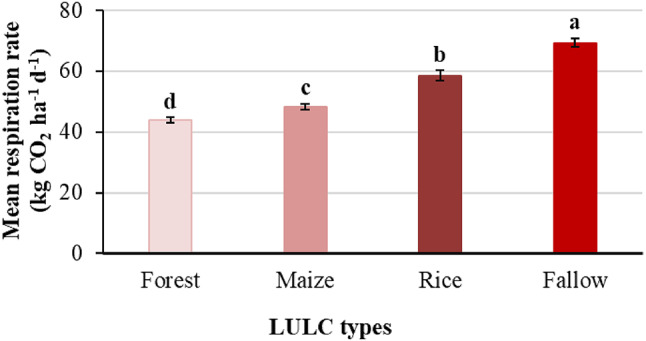
Mean respiration rate by land-use land cover type.

A multifactorial ANOVA was conducted to assess the effects of LULC, topography, and habitat type on soil respiration rates (SRR) and their interactions (Appendix 3). The results showed that LULC (F = 104.9, *p* < 0.001) and habitat type (F = 25.054, *p* < 0.001) had significant effects on SRR, indicating the influence of land-use and habitat characteristics on SRR. Pairwise comparisons revealed fallow land had the highest emissions (69 ± 0.05 kg ha^− 1^ d^− 1^), significantly exceeding forests (43.96 ± 0.88 kg ha^− 1^ d^− 1^), maize (48.13 ± 0.94 kg ha^− 1^ d^− 1^), and rice fields (58.48 ± 1.72 kg ha^− 1^ d^− 1^) (Fig. 7).

**Fig. 8:**
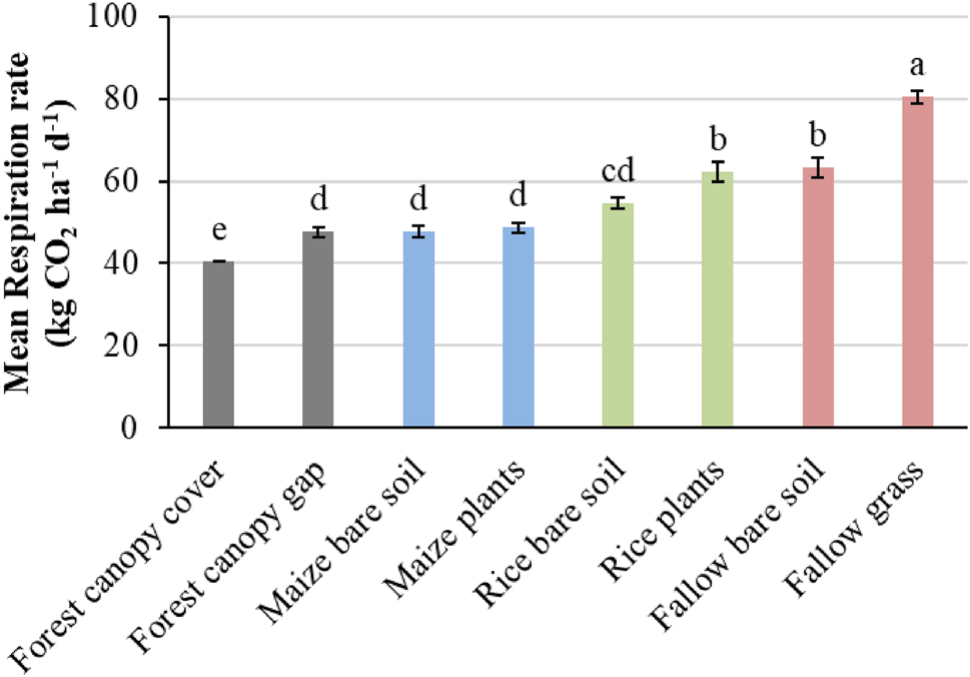
Mean respiration rate by habitat types.

The ANOVA results reveal significant differences in soil respiration rates (SRR) across habitat types within different land-use categories. The highest emissions were recorded in grass habitats.

on fallow plots, reaching nearly 80 kg ha^− 1^ d^− 1^ (Fig. 8), likely due to the abundance of plant material accelerating organic matter decomposition. In contrast, forest plots with canopy cover had the lowest respiration rate (40.46 ± 1.15 kg ha^− 1^ d^− 1^), which could be explained by limited soil moisture rather than low temperature. Throughout the study, soil temperatures in forest plots remained within 25–30 °C, while WFPS values were consistently low, mostly below 10% and approaching zero between December 2023 and March 2024, indicating severe moisture stress. This persistent deficit is likely linked to canopy interception, which reduces effective rainfall at the forest floor, and high evapotranspiration under tropical conditions. These factors together restricted microbial and root respiration, resulting in lower CO_2_ efflux from forest soils compared with other land-use types.

Intermediate emissions were observed in agricultural lands, with maize and rice fields displaying significant differences, indicating that crop types influence soil CO_2_ dynamics during cultivation. However, bare land within maize and rice fields showed no significant differences in emissions, though maize bare plots tended to emit less than rice bare plots.

Additionally, interactions between land-use type and habitat type, as well as land-use type and topography, were not significant (*p* > 0.05).

### Modelling soil respiration rate

*Relationship between soil parameters and carbon dioxide emission*.

The relationships between soil physico-chemical properties and soil respiration rate (SRR) were examined using SRR as the response variable and the measured soil parameters as predictors. Results from the correlation analysis (*n* = 104) indicate positive associations between SRR and organic matter (OM), pH, silt content, soil moisture (SM), and soil temperature (ST), with corresponding correlation coefficients of 0.43, 0.40, 0.36, 0.26, and 0.16, respectively. Sand content was negatively correlated with SRR (*r* = − 0.34). These findings suggest that multiple soil properties may be linked to observed variations in SRR within the study area.

*Stepwise regression analysis of soil respiration predictors*.

A stepwise multiple regression analysis was conducted to assess the influence of OM, pH, silt, and SM on SRR, based on the pairwise correlations in Table 4. Four regression models, including linear, logarithmic, exponential, and mixed were evaluated for their predictive performance using these edaphic factors. The mixed model outperformed all others, achieving the highest R^2^ (0.58), the lowest RMSE (8.03), and the best AIC (58.69), with minimal percent Bias value (0.0027).

These results indicate that the mixed model effectively captures the complex interactions between soil properties and SRR, making it the most reliable predictor among the models tested. Detailed regression outputs are provided in Appendix 4.

**Table 4:** Correlation matrix of soil physico-chemical parameters and soil respiration rate (SRR).

SRR	SRR	WFPS	ST	RH	AirT	BD	Porosity	pH	Phosphorus	%*N*	OM	Sand	Clay	Silt
	1	0.26	0.16	−0.16	0.21	−0.29	0.29	0.4	−0.29	−0.23	0.43	−0.34	0.18	0.36
WFPS	0.26	1	0.48	−0.43	0.61	0.18	−0.18	0.37	−0.38	−0.41	−0.26	−0.34	0.43	0.17
ST	0.16	0.48	1	−0.98	−0.97	0.27	−0.25	−0.21	−0.2	−0.49	−0.14	−0.58	0.71	0.32
RH	−0.16	−0.43	−0.98	1	0.94	−0.26	0.25	−0.23	0.22	−0.5	−0.36	0.6	−0.72	−0.34
AirT	0.21	0.61	−0.97	0.94	1	0.27	0.25	0.16	−0.28	−0.51	−0.36	0.6	−0.71	0.34
BD	−0.29	0.18	0.27	−0.26	0.27	1	−1	−0.17	0.03	0.51	−0.57	−0.17	0.13	0.15
Porosity	0.29	−0.18	−0.25	0.25	0.25	−1	1	−0.03	−0.03	−0.18	0.57	0.21	−0.13	−0.15
pH	0.4	0.37	−0.21	−0.23	0.16	−0.17	−0.03	1	−0.07	−0.18	−0.13	0.11	0.27	0.11
Phosphorus	−0.29	−0.38	−0.2	0.22	−0.28	0.03	−0.03	−0.07	1	−0.13	−0.26	0.36	−0.28	−0.31
%N	−0.23	−0.41	−0.49	−0.5	−0.51	0.51	−0.18	−0.18	−0.13	1	−0.13	−0.48	−0.39	0.03
OM	0.43	−0.26	−0.14	−0.36	−0.36	−0.57	0.57	−0.13	−0.26	−0.13	1	0.18	0.39	0.03
Sand	−0.34	−0.34	−0.58	0.6	0.6	−0.17	0.21	0.11	0.36	−0.48	0.18	1	−0.78	−0.89
Clay	0.18	0.43	0.71	−0.72	−0.71	0.13	−0.13	0.27	−0.28	−0.39	0.39	−0.78	1	0.59
Silt	0.36	0.17	0.32	−0.34	0.34	0.15	−0.15	0.11	−0.31	0.03	0.03	−0.89	0.59	1

Soil respiration rate (SRR), Water-filled pore space (WFPS), Soil temperature (ST), Relative humidity (RH), Air temperature (AirT), Bulk density (BD), percentage total nitrogen (%N), Organic matter (OM).

## Discussion

### Effect of land-use change, topography and habitat types on soil respiration rate

#### Variation of carbon dioxide across land-use types

A multifactorial analysis of variance (ANOVA) was performed to determine the effect of land-use, habitats and topography on SRR. The results reveal significant effects of land-use and habitat types on SRR, while topography had no significant influence.

Carbon dioxide (CO_2_) emission is known to vary significantly across different vegetation or land-use types^40^^,41^. In Fig. 7, the fallow plot exhibits the highest SRR and differs significantly from the other land-use types. This may be due to its higher accumulation of soil organic matter (SOM) (Table 2), which decomposes rapidly when exposed to favourable conditions such as sunlight and aeration^42^^,43^ reported that when fallow land remains uncultivated with no management practices for several years, it accumulates significant amounts of organic matter. The organic matter is then broken down by microbial activity, especially under favourable conditions of soil moisture, temperature, sunlight and aeration, leading to a sharp increase in SRR. The results of the study show that fallow land could emit more than 69 kg CO_2_ ha^− 1^ d^− 1^, which is significantly higher than the other land-use types. Furthermore, the lower bulk density of the soils in fallow plots (Table 2), as reported by, improves aeration. Such soils have reduced compaction, and enhanced microbial access to organic carbon which accelerates decomposition^44^^,45^. Consequently, microbial respiration increases, leading to greater CO_2_ release. Collectively, these findings show that fallow lands, with high organic matter content and favourable conditions for decomposition, are significant contributors to emissions from soil respiration.

In the croplands, particularly in rice fields, SRR was significantly high, although not as high as in fallow lands (Fig. 7). The distinct environmental conditions in rice fields, characterized by alternating periods of flooding and drought, create a complex dynamic that influences CO_2_ release. During flooded periods, rice fields emit primarily methane (CH_4_) due to anaerobic decomposition processes. However, during dry periods when the fields are drained, aerobic conditions prevail, leading to increased SRR^41–45^^,46^. In the current study, rice fields were managed under rainfed conditions. While it remains unclear whether anaerobic conditions were sustained throughout the wet season, the recorded maximum soil water-filled pore space (WFPS) was below 50%, suggesting that the soil remained unsaturated and retained air-filled pore spaces. This implies that conditions may not have fully favoured anaerobic decomposition processes such as methanogenesis^47^. However, the observed moisture levels during the data collection likely supported predominantly aerobic decomposition, as oxygen remained available in the unsaturated soil pores, promoting CO_2_ release over CH_4_^47,48^. Among the land-use types, maize plots exhibited lower SRR compared to fallow lands and rice fields. This could be attributed to the high BD observed in maize plots, which reduces pore space, limits aeration, and slows microbial activity, thereby inhibiting the decomposition of OM CO_2_ production^49^^,50^. Furthermore, the low organic matter and nitrogen content in the maize plots due to farm management practices likely restricted microbial substrates, further reducing SRR^45^.

Forest soil recorded the lowest SRR among the four land-use types. While their high organic matter content and low bulk density would typically promote microbial activity and soil aeration, these favourable conditions were offset by low soil moisture levels (Fig. 5). Throughout the study period, forest plots exhibited WFPS values mostly below 10%, indicating limited water availability for microbial and root respiration. This moisture constraint likely imposed physiological stress that reduced SRR, overriding the stimulatory effects of high OM and low BD. Consequently, despite their potential for active decomposition, forest soils functioned as relatively low-emitting systems under persistent moisture limitation^45^–^51^. Furthermore, the decomposition activity in forest soils can be attributed to the physiological and biochemical constraints imposed by low soil moisture^51^. Under dry conditions, microbial enzymes responsible for breaking down complex organic compounds such as lignin and cellulose become less effective, and the diffusion of soluble substrates is reduced^52^. These conditions slow the turnover of organic matter even when temperature and substrate availability are favourable. Moreover, prolonged dryness can limit root respiration and nutrient cycling, further contributing to the low SRR observed in forest plots^52^^,53^.

#### Variation of carbon dioxide across habitat types

The results from Fig. 8 reveal significant differences in soil SRR across various habitat types. These differences are influenced by vegetation type, soil conditions, and land management practices, with each habitat type exhibiting distinct emission patterns. Since an opaque chamber was used, all observed SRR are due to dark respiration, which includes both heterotrophic respiration (from soil microorganisms) and autotrophic respiration (from plant roots), but not photosynthesis^54^.

Fallow grass habitats recorded the highest levels of soil SRR, approaching 80 kg CO_2_ ha^− 1^ day^− 1^. This is likely due to a combination of significant root respiration and microbial respiration. Since no light entered the chamber, dark respiration from plant roots contributed substantially to the total SRR. Studies by ^8^ suggest that grasslands, fallows in this case, with well-established root systems, tend to exhibit higher respiration rates due to both root activity and the decomposition of organic material. Fallow bare soil and rice plant habitats showed similar emissions, slightly lower than those of the fallow grass (Fig. 8). In the fallow bare soil, SRR is primarily driven by microbial activity decomposing organic matter. In rice systems, root respiration and microbial decomposition are known to contribute to CO_2_ emissions, as reported in previous studies^47^. However, the temporary waterlogged conditions in rice fields can create anaerobic zones, limiting aerobic respiration and potentially reducing SRR compared to drier systems^47–54,54^–^55^.

Emissions from rice bare soil were significantly lower than those from fallow habitats but statistically similar to emissions from maize habitats, including maize bare soil and maize plants. This may be attributed to microbial decomposition. Although maize roots contribute to respiration, the overall root biomass and organic inputs are lower than in perennial systems like fallow grass, which recorded 1.56% and 3.48% of organic matter content respectively. Moreover, frequent disturbances in maize fields during seeding, pesticide, and applications can disrupt microbial communities, reducing SRR^56^. It is also important to note that maize plants were affected by fall armyworms and other herbivores during their vegetative stage, which occurred less than a month after seeding. Moreover, those that survived could not be monitored after 45 to 50 days of planting, as their height and width exceeded the chamber size used for measurements (25 cm diameter and up to 140 cm height). These factors likely explain the similar SRR recorded across the maize habitat types.

Forest soils under canopy cover and canopy gaps displayed the lowest SRR, particularly in canopy cover areas. Forest soils generally have slower decomposition rates due to lower temperatures and higher moisture, which reduces microbial activity^57^–^58^. Although more sunlight may stimulate root and microbial activity in the canopy gap, dark respiration measurements suggest that total root biomass may be lower than in open fallow or cropland.

#### Variation of carbon dioxide across topography

The results show that topography has a non-significant effect on soil respiration. This is even though topography is usually considered a critical factor in determining soil moisture, temperature, and organic matter distribution. This finding contrasts with earlier research by^18^, who argued that topographical variations, such as slope and elevation, create microclimatic conditions that significantly impact soil carbon cycling and microbial activity. Sloped terrains, for example, may have different moisture retention capacities and erosion patterns, which in turn affect organic matter decomposition rates and SRR^59^. Steeper slopes generally experience greater runoff, leading to reduced moisture retention and possibly lower microbial activity. However, the lack of a significant difference in SRR across topography may be due to plot selection, which was based on altitude. As shown in Appendix 1, the altitude difference between lowland and highland plots is less than 10 m on average for both maize and fallow land-use, except in the forest land. In contrast, the forest plots had an altitude difference exceeding 30 m between lowland and highland plots. This higher altitude variation within the forest plots may explain why significant differences were observed between the forest topography, while no significant difference was found in maize and fallow topography as captured in Fig. 5.

### Seasonal variation and soil respiration rate

Seasonal variations have a significant impact on SRR due to changes in environmental factors such as ST and SM, which influence biological processes, including the activity of soil microorganisms and plant roots^18^. These effects are particularly pronounced in tropical regions like Ghana, where there are distinct wet and dry seasons, with soil moisture playing a critical role.

The influence of seasonal dynamics on SRR has been clearly demonstrated in studies specific to seasonal changes in temperate, sub-tropical, and boreal regions^61^. Favourable soil moisture and temperature might lead to higher microbial and root activity as described in Appendix 3 and Table 2. The potential increase in biological activity leads to higher rates of OM decomposition and consequently, higher SRR in the wet season. Studies have shown that peak microbial activity often coincides with peak soil moisture during the wet seasons, leading to increased SRR^64^. Furthermore, the stagnation of water in the field can raise soil moisture levels, creating anaerobic areas, especially in rice fields. This can lead to increased CO_2_ production and the emission of methane and nitrous oxide^65^. Conversely, during the dry season, lower soil moisture and higher temperatures may limit microbial activity and root growth, resulting in lower SRR. This is reflected in Appendix 3, where respiration rates were generally higher during the wet season, although the difference between the two seasons was not statistically significant. There is evidence in the literature that soil microorganisms require certain levels of moisture and temperature to maintain metabolic activity. Though most existing studies are based in temperate and sub-tropical climates (e.g^67^.,), their findings remain relevant to tropical contexts. In this study, SRR slightly increased during the wet season, likely due to elevated soil moisture (SM); however, the relationship between SM, soil temperature (ST), and SRR was inconsistent across land-use types (Appendix 3, Table 2). This aligns with^51^, who found no consistent effect of land use on SRR sensitivity to ST and noted unusual patterns where ST effects were stronger under low moisture conditions^71^. similarly reported nonlinear and land-use-dependent responses of SRR to both SM and ST. In contrast^12^, observed a stronger and more consistent relationship between ST and SRR than between SM and SRR across forest, plantation, and arable land, suggesting site and method-specific variations. However, similar to this finding, they acknowledged that the precise role of SM, particularly its threshold effects on autotrophic and heterotrophic respiration, remains unclear and requires further investigation. This is especially important in tropical regions, where limited temperature variation affects decomposition and overall respiration.

### Selection of models for predicting soil respiration

In this study, SOM and pH were positively correlated with mean annual soil respiration rate (SRR) for the four land-use types (Table 2), with correlation coefficients r ≈ + 0.42. Similar observations were reported by^72^who demonstrated that higher SOM and optimal pH levels enhance microbial activity, thereby increasing SRR through the decomposition of OM^73^. also found that SOM and pH significantly influence SRR by promoting microbial respiration in tropical forest soils. Furthermore^74,75^, have observed a close link between SOM, pH, and soil SRR, as these variables support microbial metabolism and nutrient cycling essential for CO_2_ production across land-use types.

The results from the stepwise regression analyses revealed a complex interaction between SRR and the soil parameters used (Appendix 4). Although soil moisture (SM) and soil temperature (ST) are often cited as important factors influencing soil CO_2_ production and emissions^12–73,72^^,73^. The mixed models in this study captured relatively weak relationships between these variables and SRR (0.26 and 0.16, respectively; Table 4), likely due to high temporal variability, nonlinear responses, and interactions between ST and SM not accounted for by the model. Smaller sample pooling for pH, OM, silt, and sand artificially increases correlations by reducing variance, smoothing gradients, and potentially inflating correlation coefficients. Consequently, the mixed model primarily captures static spatial drivers (pH, OM, silt, and sand), while temporal stressors (SM, ST) remain masked and should be interpreted with caution. For example, although moisture content can both inhibit and stimulate SRR under tropical conditions (Fig. 4), the model only captures the overall positive effect, failing to resolve specific inhibitory and activation threshold^74^.

### Contribution to regional greenhouse gas emission calculations and Ghana’s NDCs

This study offers critical insights into soil respiration dynamics across various land-use types, providing site-specific data that can enhance Ghana’s greenhouse gas (GHG) emission calculations for the agriculture, forestry, and other land-use (AFOLU) sector. The variability in soil respiration observed in this study reflects the influence of land-use practices and soil properties on carbon fluxes, emphasizing the importance of localized data in refining emission estimates. This aligns with recent findings by^77^, who reported variations in SRR among different land-use types in the agro-pastoral ecotone of Inner Mongolia.

Ghana currently relies on IPCC Tier 1 methodologies, which use global default values for emission factors, potentially underrepresenting or overestimating the true emissions from its unique ecosystems. By integrating the emission factors from this study, it is possible to transition to Tier 3 methodologies^76^. These methods consider local conditions, such as soil properties, land management practices, and seasonal variations, which were shown to significantly influence SRR in this study. For instance, the seasonal trends in soil moisture and temperature demonstrate their critical role in driving soil carbon emissions, highlighting the need for their inclusion in refined emission models.

In the context of Ghana’s Nationally Determined Contributions (NDCs) under the Paris Agreement, these findings are particularly relevant as they provide empirical support for developing targeted mitigation strategies. These include promoting sustainable land management practices, reforestation, and optimizing agricultural inputs. For instance, the higher SRR recorded in fallow land suggests that emission reduction programs should prioritize fallow areas with fewer trees, aiming to increase tree cover. This approach would enhance soil stability and help reduce emissions^77^.

## Conclusions and way forward

The study assessed seasonal variations in soil respiration rates across four land-use types, including forest, fallow, maize, and rice in the moist semi-deciduous forest zone in Ghana. The results reveal that SRR were highest in fallow, rice, and maize, while forests recorded the lowest emissions despite their having a high soil organic matter content. This could be attributed to minimal soil disturbance in forested areas, together with the lower sunlight reaching the forest floor. These results underscore the importance of preserving forest reserves and promoting agroforestry systems to mitigate GHG emissions. The study also found that SRR from the rice paddies occurred primarily during aerobic conditions, as the soil was not fully saturated to enable anaerobic decomposition to take place, promoting methane emission. Seasonal variability significantly influenced SRR across land-use types, with higher emissions recorded during the wet season across all land-use types. However, no consistent relationship was observed between SRR and soil temperature or moisture. A regression model was developed using SRR and soil physico-chemical properties, including soil moisture, organic matter content, pH, and silt, which collectively explained approximately 58% of the observed variability in SRR.

Future research should investigate additional soil parameters, such as microbial biomass carbon and nitrogen, to better explain variations in SRR across land-use types. Furthermore, given the weak linear relationships observed in this study, future work should also examine how soil moisture and temperature influence SRR across defined threshold ranges rather than assuming linear behaviour. Identifying soil moisture and temperature categories that represent *dry*, *optimal*, or *stress* conditions would help isolate the points at which microbial and root respiration become limited. Together, these efforts will improve understanding of key controls on soil-atmosphere carbon exchange and support the development of more effective GHG management strategies.

## Data Availability

The data supporting the findings of this study are available from the corresponding author upon reasonable request.

## Appendix

Appendix tab see 1, 2 and 3, 4

**Appendix 1:** Coordinates of the selected plot for the four land categories.

Land categories	Topography	Plot	Longitude	Latitude	Altitude
Forest	Lowland	1	-1.34186	6.68722	266
Forest	Lowland	2	-1.34667	6.68767	267
Forest	Upland	1	-1.34939	6.68672	289
Forest	Upland	2	-1.34861	6.68481	309
Fallow	Upland	2	-1.36412	6.66202	254.2
Fallow	Upland	1	-1.35889	6.66889	258.3
Fallow	Lowland	1	-1.35454	6.66941	258.5
Fallow	Lowland	2	-1.36551	6.66038	263.1
CL (Maize)	Lowland	1	-1.35254	6.67146	233.1
CL (Maize)	Lowland	2	-1.34784	6.65478	249.2
CL (Maize)	Upland	1	-1.3495	6.65645	256.5
CL (Maize)	Upland	2	-1.34763	6.65484	257.1
CL (Rice)	Lowland	1	-1.36151	6.66833	249.4
CL (Rice)	Lowland	2	-1.35256	6.6719	238.4

*Note*: CL = Cropland

**Appendix 2:** ANOVA results of soil respiration rate

Source	Df	Sum Sq	Mean Sq	F value	Pr(>F)
LULC	3	209,838	69,946	104.9	2e^-16^*
Topography	1	1,916	1,916	2.999	0.08346.
Habitat type	3	48,005	16,002	25.054	6.65e^-16^*
LULC × habitat type	1	1,907	1,907	2.986	0.08416
LULC × topography	2	1,248	624	0.977	0.37668
Residuals	1953	1,247,375	639		

Significance difference between land-use types

Fallow Rice Maize Forest.

“a” “b” “c” “d”.

Significance difference across months

May24 Jun24 Mar24 Jun23 Jan24 Apr24 Oct23 Nov23 Aug23 Sep23 Jul23 Dec23 Feb24.

“a” “ab” “ab” “abc” “abc” “abcd” “bcde” “cdef” “defg” “defg” “efg” “fg” “g”.

Interaction between land-use types*months and their significance difference (letters)

Fallow: Jan24 Rice: May24 Fallow: Jun24 Fallow: Mar24 Fallow: May24 Fallow: Jul23 Fallow: Nov23.

“a” “ab” “abc” “abcd” “abcd” “abcdef” “abcde”.

Fallow: Feb24 Rice: Jun23 Rice: Jan24 Fallow: Dec23 Fallow: Jun23 Rice: Jun24 Fallow: Sep23.

“abcdef” “abcdefghijk” “abcdefghi” “abcdefg” “abcdefghi” “abcdefghij” “bcdefg”.

Fallow: Apr24 Fallow: Oct23 Maize: Oct23 Rice: Oct23 Forest: Mar24 Forest: Jun23 Rice: Apr24.

“bcdefg” “cdefg” “cdefgh” “bcdefghijk” “cdefghi” “bcdefghijk” “cdefghijk”.

Rice: Feb24 Maize: Jun23 Fallow: Aug23 Maize: Aug23 Rice: Mar24 Forest: Jun24 Maize: Jun24.

“cdefghijk” “cdefghijk” “defghijk” “cdefghijklm” “cdefghijklm” “efghijk” “efghijk”.

Maize: May24 Rice: Nov23 Rice: Dec23 Maize: Mar24 Maize: Jul23 Forest: Apr24 Maize: Apr24.

“efghijk” “defghijklm” “cdefghijklm” “efghijk” “efghijklm” “efghijkl” “efghijk”.

Rice: Aug23 Forest: Nov23 Forest: Jan24 Maize: Jan24 Forest: May24 Maize: Sep23 Forest: Sep23.

“efghijklm” “efghijklm” “fghijk” “efghijklm” “fghijklm” “ghijklm” “ijklmn”.

Rice: Sep23 Maize: Nov23 Forest: Aug23 Maize: Dec23 Forest: Oct23 Forest: Dec23 Rice: Jul23.

“ghijklmno” “hijklmno” “hijklmno” “jklmno” “klmno” “lmno” “hijklmno”.

Forest: Feb24 Maize: Feb24 Forest: Jul23.

“mno” “no” “o”.

**Appendix 3:** Seasonal variation of soil respiration rate and related soil moisture across land-use types.

		Fallow			Forest			Maize			Rice		
Seasons	Months	SRR kg ha^-1^d^-1^	SM (%)	ST (℃)	SRR kg ha^-1^d^-1^	SM (%)	ST (℃)	SRR kg ha^-1^d^-1^	SM (%)	ST (℃)	SRR kg ha^-1^d^-1^	SM (%)	ST (℃)
Wet	Jun23	64.59	19.13	29.30	58.52	9.58	27.43	57.17	25.43	29.50	66.40	35.65	30.91
	Jul23	70.78	13.20	28.75	17.46	14.10	26.09	51.70	16.98	29.61	31.46	45.38	28.74
	Sep23	64.27	15.62	27.97	41.77	14.57	25.61	45.85	18.09	31.11	41.42	38.73	30.83
	Oct23	63.99	14.21	29.25	36.29	13.69	26.40	59.79	14.28	33.13	59.41	34.73	32.88
	Nov23	67.93	11.96	28.64	50.03	9.37	26.92	40.95	19.02	32.48	53.72	29.52	33.18
	Apr24	64.19	3.85	33.33	51.08	2.88	29.31	50.62	3.53	37.18	58.31	19.10	37.11
	May24	76.43	7.05	30.75	46.25	6.09	26.82	53.76	8.33	35.27	85.80	16.74	35.54
	Jun24	78.43	9.30	28.80	54.01	6.32	26.57	53.93	9.87	32.18	64.30	26.97	32.91
	Mean	68.83^a^	11.79^a^	29.60^a^	44.43^a^	9.58^a^	26.89^a^	51.72^a^	14.44^a^	32.56^a^	57.60^a^	30.85^a^	32.76^a^
Dry	Aug23	56.12	14.94	27.71	40.91	15.13	25.03	55.64	20.99	27.69	50.51	39.16	27.93
	Dec23	65.46	6.75	27.91	33.52	3.36	25.43	37.91	7.41	37.19	53.48	24.02	34.55
	Jan24	88.36	7.45	30.08	49.26	0.36	28.21	48.70	9.69	38.09	65.54	17.08	34.30
	Feb24	67.40	3.94	38.73	28.99	0.00	29.42	24.56	5.87	37.70	57.50	14.49	32.64
	Mar24	77.24	5.73	33.68	58.82	1.28	30.29	53.44	5.70	37.15	54.89	22.72	32.66
	Mean	70.91^a^	7.76^b^	31.62^a^	42.30^a^	4.03^b^	27.68^a^	44.05^b^	9.93^b^	35.56^a^	56.38^a^	23.49^b^	32.41^a^

**Appendix 4:** Stepwise non-linear multiple regression models of soil respiration rate on edaphic variables.

Models	Variables	Multiple Regression Model	*R* ^2^	RMSE	AIC	%Bias
Linear	OM	$\:SR=44.583+3.607OM$	0.152	11.17	212.93	0.00
Linear	OM, pH	$\:SR=-23.596+4.087OM+11.778pH$	0.339	9.66	207.09	0.00
Linear	OM, pH, Silt	$\:SR=-25.741+3.988OM+10.889pH+0.705Silt$	0.408	8.95	204.96	0.00
Linear	OM, pH, Silt, SM	$\:SR=-21.652+4.134OM+9.589pH+0.657Silt+0.185SM$	0.403	8.79	205.99	0.00
Logarithmic	OM	$\:SR=46.630+8.963log\left(OM\right)$	0.144	11.44	214.25	0.00
Logarithmic	OM, pH	$\:SR=-74.982+11.249\:log\left(OM\right)+69.037log\left(pH\right)$	0.377	9.76	207.67	0.00
Logarithmic	OM, pH, Silt	$\:SR=-78.030+11.962\:log\left(OM\right)+60.840\:log\left(pH\right)+7.540log\left(Silt\right)$	0.509	8.66	203.21	0.00
Logarithmic	OM, pH, Silt, SM	$\:SR=-75.134+12.311\:log\left(OM\right)+52.862\:log\left(pH\right)+6.569\:log\left(Silt\right)+4.558\:log\left(SM\right)$	0.530	8.48	204.05	0.00
Exponential	OM	$\:{SR=45.331exp}^{\left(0.0652OM\right)}$	0.195	11.10	212.58	-0.03
Exponential	OM, pH	$\:{SR=54.0392exp}^{(0.0957OM+0.086pH)}$	0.358	9.91	208.48	0.07
Exponential	OM, pH, Silt	$\:{SR=53.7944exp}^{(0.097OM+0.094pH+0.072Silt)}$	0.473	8.98	205.14	0.01
Exponential	OM, pH, Silt, SM	$\:{SR=53.7554exp}^{(0.103OM+0.083pH+0.067Silt+0.034SM)}$	0.495	8.79	206.01	0.01
Mixed	OM	$\:SR=45.3313\:{exp}^{\left(0.0663\:OM\right)}$	0.20	11.10	58.60	0.03
Mixed	OM, pH	$\:SR=\left(0.856\:{exp}^{\left(0.0663\:OM\right)}\right)(55.9370\mathrm{log}\left(pH\right)-44.0498\left)\right)$	0.39	9.69	57.77	-0.04
Mixed	OM, pH, Silt	$$\begin{gathered} \:SR = (0.\:0161exp^{{\left( {0.0741\:OM} \right)}} )(55.9370{\mathrm{log}}\left( {pH} \right) \hfill \\ - 46.3181)(7.8634{\mathrm{log}}\left( {Silt} \right) + 36.9390) \hfill \\ \end{gathered}$$	0.56	8.24	56.50	0.03
Mixed	OM, pH, Silt, SM	$$\begin{gathered} \:SR = (0.\:000377exp^{{\left( {0.0771\:OM} \right)}} ) \hfill \\ (55.9370{\mathrm{log}}\left( {pH} \right) - 36.2977) \hfill \\ (6.3644{\mathrm{log}}\left( {Silt} \right) + 36.9390) \hfill \\ (3.2327{\mathrm{log}}\left( {SM} \right) + 24.48) \hfill \\ \end{gathered}$$	0.58	8.03	58.69	0.00
